# MENTAL HEALTH FIRST AID TRAINING: EVIDENCE OF ITS EFFECTIVENESS FOR MENTAL HEALTH PROMOTION AMONG OLDER ADULTS

**DOI:** 10.1093/geroni/igad104.0992

**Published:** 2023-12-21

**Authors:** Iftekhar Amin

**Affiliations:** University of North Texas at Dallas, Dallas, Texas, United States

## Abstract

**Background:**

About 15% of adults aged 60 and over have a mental health disorder. However, stigma and lack of knowledge of mental health resources act as barriers for them to seek professional help. Mental Health First Aid (MHFA) is an evidence-based program that teaches participants how to identify, understand and respond to signs of mental illnesses and substance use disorders. The Older Adults version of this training focuses on the unique experiences and needs of older adults. This study evaluated the effectiveness of a Substance Abuse and Mental Health Services Administration (SAMHSA) funded MHFA training program among older adults. Method: Data were collected from MHFA training sessions delivered to 27 staff and 36 residents at two assisted living facilities from January to March 2023. A quasi-experimental, pre-post design was used. Two outcomes mental health literacy and stigma were measured at the pretest, posttest, and 4 months after the the training. A paired sample t-test was performed to test the differences between the pre- and post-test.

**Results:**

Statistically significant improvements in self-confidence in promoting help-seeking and reduction in stigmatizing attitudes were observed among participants in post-tests and 4-month tests. Participants reported increased knowledge in how to provide initial support to someone who may be mental health crisis and help connect them to the appropriate care.

**Conclusion:**

Results indicate MHFA is likely to improve mental health awareness and reduce stigma among older adults. This study adds new evidence to the literature on the MHFA program’s effectiveness among older adults and their caregivers.

